# Genetic predisposition to unwanted side effects under antidepressants and antipsychotics: a molecular-genetic study of 902 patients over 6 weeks

**DOI:** 10.1007/s00406-025-02062-4

**Published:** 2025-07-28

**Authors:** Hans H. Stassen, S. Bachmann, R. Bridler, K. Cattapan, A. M. Hartmann, D. Rujescu, E. Seifritz

**Affiliations:** 1 Institute for Response-Genetics, Department of Psychiatry, Psychotherapy and Psychosomatics, Psychiatric University Hospital, 8032 Zurich, Switzerland; 2 Department of Psychiatry, Geneva University Hospitals, 1226 Thônex, Switzerland; 3 Sanatorium Kilchberg, 8802 Kilchberg, Switzerland; 4 University Hospital of Psychiatry and Psychotherapy, University of Bern, 3012 Bern, Switzerland; 5 Clinical Division of General Psychiatry, Medical University of Vienna, 1090 Wien, Austria; 6 Department of Psychiatry, Psychotherapy and Psychosomatics, Psychiatric University Hospital, 8032 Zurich, Switzerland; 7 Institute for Response-Genetics Psychiatric University Hospital (KPPP), University of Zurich, c/o Sanatorium Kilchberg, Alte Landstrasse 70, 8802 Kilchberg, Switzerland

**Keywords:** Antidepressants, Antipsychotics, Depression, Schizophrenia, Polypharmacy, Side effect profiles, Gene vectors, Genotypic patterns, Genetic diversity, Molecular-genetic neural Nets

## Abstract

This project aimed at (1) detailing the complex side effect patterns of 902 inpatients treated for major depression or schizophrenia under polypharmacy regimens; (2) developing a quantitative side effect model that accounts for the various facets of clinically observable adverse events; and (3) analyzing irregularities in genetic diversity through multidimensional “gene vectors” in order to reveal possible interrelations with side effect clusters. The patients’ acute medication, their time course of recovery, and their side effects were assessed by up to 8 repeated measurements. The genotyping included 100 candidate genes with genotypic patterns computed from 549 Single Nucleotide Polymorphisms (SNPs). Between 61.9% and 68.1% of study patients reported moderate to severe side effects, while response rates were with 29.5–35.7% quite modest. Half of the patients (52.1%) experienced weight gains of ≥ 2 kg. On the phenotype level, up to 30% of the observed variance could be “explained” by regression models with the dominating factor “number of concurrent drugs”. On the genotype level, we relied on standard Artificial Intelligence (AI) procedures along with multilayer Neural Nets (NNs) to search for combinations of multidimensional genotypic patterns that were characteristic of patients with severe side effects, while being rare (< 10%) among patients without side effects. These analyses failed to explain a clinically relevant proportion of the observed phenotypic variance. The 14 cytochromes analyzed were found to play no more than a minor role. While type and severity of side effects under polypharmacy were primarily determined by the overall medication “load”, the actually observed side effect patterns varied considerably between patients receiving the same medication “load”, thus stressing the role of genetics. Our results suggested that the role of genetics in the development of severe side effects under polypharmacy is by far more complex than previously assumed, related to a completely different set of genes, or that there exists genotypic heterogeneity such that multiple pathways on the genotype level lead to the same clinical picture on the phenotype level.

**Supplementary Information:**

The online version contains supplementary material available at 10.1007/s00406-025-02062-4.

## Introduction

Today, more than 90% of inpatients hospitalized for major depression or schizophrenia are treated with psychotropic drugs. As there is little proven knowledge about etiology and pathogenesis of psychiatric disorders, psychotropic drugs *are all non-causal and act in an unspecific way by triggering the onset of recovery* [1–5]. Lacking causal treatment strategies, the majority of patients receive combinations of several antidepressants, antipsychotics, mood stabilizers, anxiolytics, hypnotics, antihistamines, and anticholinergics, along with other somatic treatments (“polypharmacy”) —very much in the sense of trial and error, or by rules that are only loosely defined [1–5]. Monotherapy and psychotherapy without supplemental psychotropic medication are no longer treatment options. Not surprising at all, response rates are modest (in the range of 35-45%), while the time courses of recovery are very heterogeneous. About one third of patients get “stuck” after initial improvement, and some 20% do not show any improvement at all. In schizophrenia, antipsychotics reduce the positive symptoms very quickly, whereas general psychopathology and negative symptoms are only partially alleviated [6].

In tandem with drug treatment the burden of side effects arises, including major cardiovascular and neurological disturbances, along with impairments of sleep and excessive weight gain [7–14]. Between 50% and 70% of patients experience such troublesome and annoying side effects. No reliable prediction can be made as to (1) if and what side effects a particular patient will develop under a given treatment regimen; and (2) if and when a particular patient will respond to a particular treatment. Unwanted side effects seem to evolve in a similar way across diagnoses and substances, suggesting the existence of an unspecific and presumably genetically predisposed risk of developing such highly undesirable effects under polypharmacy.

As to medication-induced weight gain, our explorative study of 832 inpatients with ICD-10 diagnoses of either F2 (Schizophrenia; *n* = 282) or F3 (Major Depression; *n* = 550), and complemented by the data of 3,180 students, had revealed that 47.7% of F2 patients and 54.9% of F3 patients showed a weight gain of 2 kg and more after 3 weeks of treatment. This weight gain was similar across all the different substance combinations observed with the polypharmacy-oriented treatments [14]. Major predictive factors were “starting weight”, “number of concurrent medications”, and “increased appetite”. Moreover, the student data made it clear that overweight and obesity typically begin early in life among those affected, and are interconnected with personality traits, while increasing the risk of developing psychosomatic disturbances, mental health problems, or somatic illnesses [14].

A large amount of clinical data suggest that side effects under polypharmacy may be comprised of two parts: (1) a medication-independent general component that evolves in a similar way across gender, diagnoses and substances, with clinical severity being mainly determined by the patients’ overall medication “load”; and (2) a treatment-specific component that depends quite strongly on the combination of substances and their interactions. Most notably, the between-patient differences in side effects appear to be driven, at least in part, by genetic factors, *as patients receiving a similar or even an identical medication “load” can display quite different side effect patterns*,* both in terms of the type of symptoms they experience and the overall clinical severity*.

Patients may therefore have a latent physical-genetic disposition that enables, facilitates, impedes, or prevents certain side effects, and might enable a reliable prediction of side effects in the individual patient. However, genetic studies in the field of major psychiatric disorders over the past decades have not yet led to the hoped-for breakthrough, neither by means of polygenic models based on “clinical diagnosis” as phenotype, nor by Genome-Wide Association Studies (“GWASs”). Particularly disillusioning is the fact that GWAS results produced with gigantic effort explain far less than 10% of the observed phenotypic variance [15–23].

Standard genetic methods aiming at predicting response under psychopharmacological treatment [24–30], or to predict excessive medication-induced weight gain [31–34], have also not been particularly successful to date, as the candidate genes under investigation typically explained only small proportions of the phenotypic variance, while candidate SNPs were rarely ever found to be reproducible [35–41].

Consequently, this project did not rely on standard genetic association methods, nor did it aim to fit a causal polygenic model to clinical data when searching for genetically predisposed “side effect risks”. Rather, the interest was focused on *irregularities in genetic diversity* which were recently found to be closely linked to a latent physical-genetic “recovery disposition” that enables, facilitates, impedes, or prevents recovery from major psychiatric disorders by setting various thresholds for exogenous triggers to initiate improvement [42].

Specifically, we were interested in the extent to which irregularities in genetic diversity might contribute to (1) a general unspecific disposition to developing side effects under polypharmacy; (2) the development of treatment-specific side effects, such as neurological and cardio-vascular disturbances; and (3) the development of unwanted medication-induced weight gain.

Genetic diversity can be assessed through multidimensional “gene vectors” [21, 42], so that systematic analyses of the genotypic patterns inherent in the patients’ “gene vectors” will reveal any irregularities in genetic diversity. The “gene vectors” are typically assembled from 4 to 8 polymorphic SNPs located within genes and representing the genes’ distinctive “fingerprints”. As SNPs can exhibit three different expressions (regardless of allele definition), each SNP can take on one of three different binary patterns: 0/0, 0/1, or 1/1.

With ***m*** SNPs, a total of 3^***m***^ different genotypic patterns is theoretically observable per gene vector: 81 patterns for 4 SNPs, 243 patterns for 5 SNPs,…. 6,561 patterns for 8 SNPs. The minimum sample size required to actually observe that number of different genotypic patterns can be estimated. Under the assumption of equally distributed SNPs, a sample size of 4 times 3^***m***^ is expected to exhibit about 95% of the theoretically possible patterns.

The resulting multidimensional genotypic patterns can then be evaluated by machine learning techniques following a two-stage approach: (1) using standard Artificial Intelligence (AI) procedures to screen the genes under investigation for multidimensional genotypic patterns that are characteristic of patients with side effects, while being rare (< 10%) among patients without side effects; (2) genes that meet these criteria (“side effect genes”) will get the highest weight in subsequent Neural Net analyses (“NNs”).

In conclusion, we aimed at (1) detailing the complex side effect patterns observed among inpatients with major depression or schizophrenia under polypharmacy; (2) developing a comprehensive quantitative side effect model that accounts for the various facets of clinically observable adverse events; and (3) analyzing irregularities in genetic diversity as provided by multidimensional “gene vectors” to detect possible interrelations with side effect clusters.

## Materials and methods

In a “naturalistic” observational study of psychiatric inpatients, we recruited 902 patients hospitalized at five residential mental health treatment centers with an ICD-10 diagnosis of either schizophrenic (*n* = 264; “F2 patients” [143 males, 121 females]) or depressive disorders (*n* = 638; “F3 patients” [209 males, 429 females]). New admissions with a suspected primary ICD-10 diagnosis of “F2x.x” or “F32.x/F33.x” were invited to enroll in the study. All participating patients signed a written “informed consent” after having been informed about the aims of the project, and that they can stop their participation at any time without any disadvantages. Final diagnoses were decided by two senior psychiatrists.

The study protocol included (1) up to 8 repeated measurements over 6 weeks assessing the time course of improvement through the 17/21-item Hamilton Depression Scale HAMD [43] or the 30-item Positive and Negative Syndrome Scale PANSS [44]; (2) up to 8 repeated measurements over 5 weeks assessing medication and unwanted side effects through the 46-item Medication and Side Effects Inventory MEDIS [45]; and (3) the collection of blood samples for serum extraction and DNA isolation. Psychopathology was assessed by specifically trained psychiatrists and psychologists to improve inter-rater agreement.

A minimum baseline score of at least 15 on the HAMD17 Scale (primary “F32.x/F33.x” diagnoses), or of at least 21 on the general psychopathology PANSSG Scale (primary “F2x.x” diagnoses), was required at entry into study. The PANSSG scale was chosen in order to prioritize illness-related disabilities in daily functioning over acute productive symptomatology and longer persisting negative symptoms. Baseline severity was categorized in the following way; among F3 patients: HAMD17 < 20 as “mild”; HAMD17 from 20 to 24 as “moderate”; and HAMD17 > 24 as “severe”; among F2 patients: PANSSG < 30 as “mild”; PANSSG from 30 to 40 as “moderate”; and PANSSG > 40 as “severe”. In line with our previous studies in this field [2, 3, 42] we used scale-based cutoff values for the definition of response to treatment. “Response” under schizophrenia therapies was defined by a sustained 40% PANSSP baseline score reduction and under depression therapies by a sustained 50% HAMD17 baseline score reduction^1^. Similarly, we defined “onset of improvement” by a sustained 20% PANSSP, or a 20% HAMD17 baseline score reduction, respectively.

The 48-item MEDIS instrument details the following side-effect clusters: «sleep» (5 items), «appetite» (3 items), «sexuality» (3 items), «gastro-intestinal» (7 items), «cardiac-respiratory» (4 items), «autonomic» (5 items), «psychosomatic» (6 items), «neurological» (9 items) and «cardio-vascular» (4 items). The patients’ response to therapeutic interventions was assessed through a longitudinal profile encompassing up to 8 repeated HAM-D, PANSS, and MEDIS scores regarding medications and side effects. The global side effect score S was stratified as follows: (1) no side effects: S ≤ 10; (2) mild side effects: 10 < S ≤ 30; (3) moderate side effects: 30 < S ≤ 40; (4) severe side effects: 40 < S ≤ 50; and (5) very severe side effects: 50 < S. The side effect scores derived from the side-effect clusters were stratified analogously.

The patients’ weight gain was determined after 3 weeks of treatment. Patients, who dropped out prematurely but had weight measurements after 2 weeks, were also included in the analysis by means of the LOCF method (Last Observation Carried Forward). As the weight fluctuations among 577 control subjects were in the range of ± 1.2 kg (two standard deviations) at 14-day intervals, we defined changes of ≥ 2 kg as significant “weight gain”, or “weight loss”^2^. Patients described a weight gain of ≥ 2 kg as clearly noticeable, unpleasant, and quite irritating.

### Ethics

The study was approved by the local ethics committees in charge of the participating centers. Written informed consent was obtained from all participants.

### Statistical analyses

We used the Statistical Analysis Software SAS/STAT 9.4 by SAS Institute Inc. for repeated measurement analyses (PROCs ANOVA, CORR(PEARSON/SPEARMAN), FREQ(CHISQ), GLM, NPAR1WAY, TTEST), and the SPSS 28 Statistics Package by IBM, along with PROC *HPNEURAL* from SAS Enterprise Miner 15.1, for Neural Nets analyses.

### Genotyping

Critically important for the envisaged analyses of genetic diversity is the selection of informative genes whose “gene vectors” and genotypic patterns derived from them (1) possess sufficient variation across subjects so that subtle between-subject differences can be resolved; and (2) provide access to the postulated irregularities in genetic diversity. Due to lack of suitable a priori knowledge, the gene selection for this study was necessarily a kind of “blind shot in the dark” and mostly included genes reported in the literature as “possibly” involved in the pathogenesis of psychiatric disorders (without the existence of reliable probability estimates). Luckily enough, the selection of the 100 candidate genes in preparation for this study included quite a number of highly informative genes, so that the study could be completed successfully. The genes’ genotypic patterns were assessed through 549 Single Nucleotide Polymorphism (SNPs).

Genotyping was performed using the iPLEX assay on the MassARRAY MALDI-TOF mass spectrometer “Sequenom” [46], multiplexed with 40 + separate loci per reaction. This method is based on single base extension (SBE) of SNP specific primers using mass modified ddNTPs. In addition, SBE primer length was used to ensure unambiguous resolution of SNPs and alleles. Quality criteria were a sample call rate > 80%, SNP call rate > 95%, and genotypes of CEU Trios in accordance with HapMap database > 98%.

### Quantifying genetic diversity

The quantification of genetic diversity inherent in a population relied on “gene vectors” which were assembled per gene from the genotypes of 4–8 polymorphic SNPs located within each gene. As a SNP can exhibit three different expressions regardless of allele definition, a base-3 system was used to construct gene vectors:

**Table:** 

“gene vector”:	$\:{v}_{i}^{\left(j\right)}=\sum\:_{k=1}^{m\left(j\right)}{s}_{ik}^{\left(j\right)}{3}^{k-1}$	*i = 1*,*2*,*… N* subjects *j = 1*,*2*,*… M* genes $\:{s}_{ik}^{\left(j\right)}$∈*{0*,*1*,*2}* SNPs *m(j)* number of SNPs in the *j-th* gene

With ***m*** SNPs, a total of *3*^***m***^ different genotypic patterns would be theoretically possible per gene. Under the assumption of equally distributed SNPs, a sample size of 4 times *3*^***m***^ is expected to exhibit about 95% of the theoretically possible patterns. This estimate was almost perfectly confirmed by simulations based on the study’s genes and SNP selections. The number of different genotypic patterns observed with “gene vectors”, the genes’ so-called “diversity index”, served as estimate of a population’s inherent genetic diversity.

### Neural Nets

**Fig. 1:**
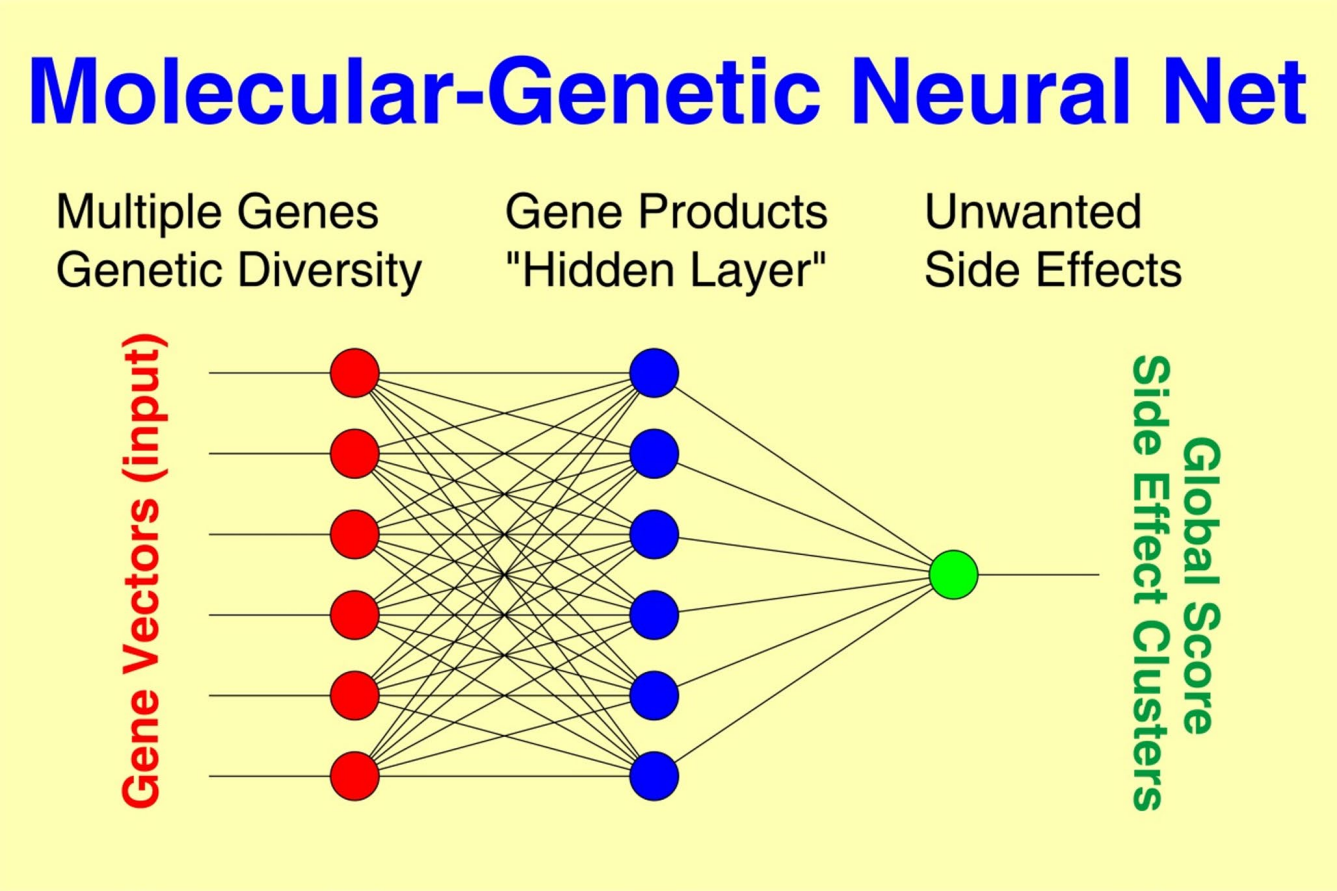
Principal schema of a multilayer Neural Net (NN) where global side effect score and side effect clusters (output) are predicted from multiple gene vectors (input) connected to each other by complex interactions via one or more “hidden” layer(s). The NN algorithm iteratively constructs a model that is simultaneously fitted to the observed data of all patients. The achievable goodness of fit depends on the information included, the quality of underlying data, and the number of intermediate layers implemented to model nonlinear interactions

Nonlinear Neural Nets (NN) connect the “neurons” of input and output layers via one or more “hidden” layers (Fig. 1), thus featuring a relatively large number of free parameters.

NN connections are realized through (1) weight matrices and (2) model fitting algorithms minimizing an error function in the weight space (goodness of fit). All outputs are computed using sigmoid thresholding of the scalar product of the corresponding weight and input vectors. Outputs at stage “***s***” are connected to each input of stage “***s*** + 1”. The most popular model fitting strategy, the backpropagation algorithm, looks for the minimum of the error function using the method of gradient descent (“steepest descent”). The basic algorithm is [47]:

**Table:** 

(i)	Output:	${s_i}=\sigma \left[ {\sum\limits_{j}^{{}} {{w_{ij}}{s_j}} } \right]$	s_i_: y_i_ observed	(i = 1,2,… *N*_i_)
(j)	Hidden layers:	${s_j}=\sigma \left[ {\sum\limits_{k}^{{}} {{w_{jk}}{s_k}} } \right]$		(*j = 1*,*2*,*… N*_j_)
(k)	Input:	${s_k}={x_k}$	*x*_*k*_ observed	(*k* = *1*,*2*,*… N*_k_)
	Improvements:	$\Delta {w_{ij}}=\alpha \cdot \varepsilon _{i}^{\nu } \cdot {s_j} \cdot {s_i}(1 - {s_i})$	$\varepsilon _{i}^{\nu }=y_{i}^{\nu } - s_{i}^{\nu }$	(*ν* = *1*,*2*,*. p*)
		$$\Delta {w_{jk}}=\alpha \cdot \sum\limits_{{i=1}}^{{{N_i}}} {\varepsilon _{i}^{\nu }} \cdot {s_k} \cdot {s_i}(1 - {s_i}) \cdot {w_{ij}} \cdot {s_j}(1 - {s_j})$$		

where ***x***_k_ denote observed stimuli, ***y***_j_ observed responses, σ the activation function of sigmoid-type: R→(0,1), ***α*** the learning rate, and ***p*** the number of probes (patients). The achievable precision of the model depends on the information included, the quality of underlying data, and the number of intermediate layers implemented to model nonlinear interactions.

Results derived through standard NN approaches, which use 80% of samples for training and the remaining 20% for testing tend to be over-optimistic, in particular in the presence of assessment errors and missing data. By contrast, the ***k***-fold cross-validation approach splits the data into ***k*** roughly equal parts, using ***k***-1 partitions for training, while one partition is used for testing. This process is repeated until each partition has served as a testing set, so that ***k*** estimates of prediction errors are generated. The resulting prediction errors are approximately unbiased for the “true” error for sufficiently large ***k*** (***k*** ≈ 10 is a typical value in practice). In consequence, we relied on the ***k***-fold cross-validation strategy with ***k*** = 10 throughout the entire project and applied the well-proven “random walk” strategy in order to distinguish between local and global minima.

## Results: phenotype level

### Response to treatment

Of the initial 1,229 patients enrolled in this study, 902 met the inclusion criteria and had at least three assessments within the first two weeks of treatment (F2 patients: *n* = 264; F3 patients: *n* = 638). Where absolutely necessary, missing assessments were filled in using standard LOCF methods (*last observation carried forward*). The response characteristics were very similar across the two diagnostic groups: only a minority of patients met the response criteria (F2 patients: 29.5%; F3 patients: 35.7%), a lot of patients got “stuck” after initial improvement, and a considerable number of patients showed no improvement at all (18.2-29.1%).

### Unwanted side effects

Monotherapy was only a treatment option at the very beginning of the study. Thereafter, the clinical reality has shifted towards ubiquitous “polypharmacy” regimens, that is, the patients were treated with a combination of several antidepressants, antipsychotics, mood stabilizers, anxiolytics, hypnotics, antihistamines, and anticholinergics, along with other somatic treatments. In consequence, the average patient of this study received 4.50 ± 2.68 medications, consisting of 3.30 ± 1.84 psychotropic drugs, plus 0.79 ± 1.13 medications that alleviate adverse side effects, plus 0.41 ± 0.89 other somatic medications. This meant, on average, 4 direct sources contributing to potential side effects plus 6 indirect sources due to various interactions between substances. The patients’ multifaceted response to polypharmacy treatment included simultaneous contributions to all side-effect clusters with frequency and severity primarily depending on the overall medication “load”.

A clear and widely accepted strategy was not discernible. Rather, treatment strategies varied not only from hospital to hospital, but also from ward to ward, defined autonomously by each ward’s senior psychiatrist. The only recognizable common ground appeared to be that F2 patients were treated with two antipsychotics plus one antidepressant, and F3 patients with two antidepressants plus one antipsychotic. Almost half of both F2 and F3 patients were treated in this way. The addition of benzodiazepines as an adjunct treatment was another commonly used approach, intended to augment the efficacy of the primary medication. This option was chosen in almost one third of cases. Even patients with mild depression (HAM-D17 baseline score of 15–19) were generally treated the “polypharmacy-oriented way” even though placebo-controlled drug trials never showed superiority of active compounds over placebo for these patients [2–4]. Another noteworthy observation was that drug-naïve patients were hardly ever seen anymore. Contributing to a good deal to this development was certainly the widespread pre-treatment of patients with antidepressants and antipsychotics by the family doctors.

Most patients experienced significant unwanted side effects induced by their medications. These side effects developed from the very beginning of treatment and were almost unchanged on day 10 of treatment. In terms of global side effect scores, as many as 87.3% of the F2 patients and 83.5% of the F3 patients reported at least “some” mild side effects. The percentages of the severe forms were 39.4% (F2) and 37.1% (F3), and for the mild to moderate forms 47.9% (F2) and 46.4% (F3). The differences between the diagnostic groups were small and did not reach statistical significance (*p* = 0.3373). However, females reported more severe side effects than males (42.1% vs. 32.1%; *p* = 0.0025; [***t***=-3.08, ***df*** = 124]). This was most likely due to the fact that females received more concurrent medications than males (4.86 ± 2.50 vs. 4.04 ± 2.43).

In order to detail the observed gender differences, we computed for all side-effect clusters the percentages of patients with moderate to severe side effects, separately for males and females. The resulting percentages are given in Table 1, sorted in ascending order.

The observed gender differences were statistically significant (*p* = 0.0048; [***chi2*** = 22.1, ***df*** = 8]), with females experiencing consistently more side effects. Hence, the observed frequencies run virtually *parallel* for males and females. The only exception was the side-effect cluster “*Sexuality*”, shaded gray in Table 1. As females received significantly more concurrent medications than males, we analyzed the extent to which the observed gender differences were due to the females’ higher overall medication “load”. Controlling for the overall medication “load” reduced the gender differences considerably so that the statistical significance disappeared when the side-effect cluster “*Sexuality*” was blanked out (*p* = 0.1194; [***chi2*** = 11.5, ***df*** = 7]). Breaking the data further down by diagnosis left the results essentially unchanged.

All this underlined the key role of the overall medication “load” when it comes to “explaining” adverse side effects in today’s psychiatry. Other factors such as “gender” appeared to play a minor role, if at all. This finding is in perfect agreement with the results of generalized linear regression models shown in a later paragraph, where the overall medication “load” was found to explain some 30% of the empirical variance inherent in the side-effect clusters.

In line with expectations, the global side effect score was found to be closely linked with the number of concurrent medications (*r* = 0.24082; *p* = 0.0005). It was therefore not surprising that patients under monotherapy experienced only a fraction of the side effects compared to patients under polypharmacy (*p* < 0.0001; [***t***=-4.03, ***df*** = 318]). For example, in the case of neurological disturbances, just 1 patient (5.3%) under monotherapy compared to 103 patients under polypharmacy (35.3%), in the case of cardiovascular disturbances just 2 patients (10.5%) under monotherapy compared to 97 patients under polypharmacy (33.2%), and regarding the global side effect score just 2 patients (10.5%) under monotherapy compared to 117 patients under polypharmacy (40.1%).

For the five side effect clusters «*Sleep*», «*Appetite*», «*Gastro-intestinal*», «*Cardiac-respiratory*», and «*Psychosomatic*», the analysis revealed an overall picture of similar side effect profiles across diagnoses and across the various polypharmacy-oriented medication regimens, thus suggesting the existence of a unspecific general predisposition to developing unwanted side effects. The five clusters were assessed through 25 items of the MEDIS instrument and developed from the very beginning of medication. The clinical severity of all clusters was only slightly reduced after 10 days of treatment (Table 2).

By contrast, the four side effect clusters «*Sexuality*», «*Autonomic*», «*Neurological*», and «*Cardiovascular*» displayed significant differences between diagnoses, presumably due to different medication priorities in the treatment of patients with F2 diagnoses (antipsychotic-oriented with antidepressants as co-medication) versus F3 diagnoses (antidepressant-oriented with antipsychotics as co-medication) (Table 3). These differences were particularly evident in (1) the «neurological» side effect cluster with 72.1% (F2) versus 39.9% (F3) [items: *hypertonia*, *hypotonia*, *tremor*, *acute dyskinesia*, *hypokinesis*, *akathisia*, *ataxia*, *nystagmus*, and *paresthesia*], and (2) the «cardiovascular» side effect cluster with 90.8% (F2) versus 72.3% (F3) [items: *orthostatic hypotension*, *hypertension*, *dysrhythmia*, and *changes of blood count*].

The four side effect clusters were assessed by the MEDIS instrument through 21 items and developed from the very beginning of medication. The clinical severity of the clusters was quite stable over time and seemed to be virtually unchanged on “*Day 10*” of the study when compared with the first assessment “*Day 01*” (Table 3).

The above findings suggested that side effects under polypharmacy treatment are comprised of two parts: (1) an *unspecific general component* evolving in a similar way across gender, diagnoses and polypharmacy-oriented substance combinations, while encompassing the symptom clusters «*Sleep*», «*Appetite*», «*Gastro-intestinal*», «Cardiac-respiratory», and «*Psychosomatic*»; and (2) a *number of treatment-specific components* that depended quite strongly on the chosen combination of substances and their interactions, while encompassing the symptom clusters «*Sexuality*», «*Autonomic*», «*Neurological*», and «*Cardio-vascular*».

**Fig. 2:**
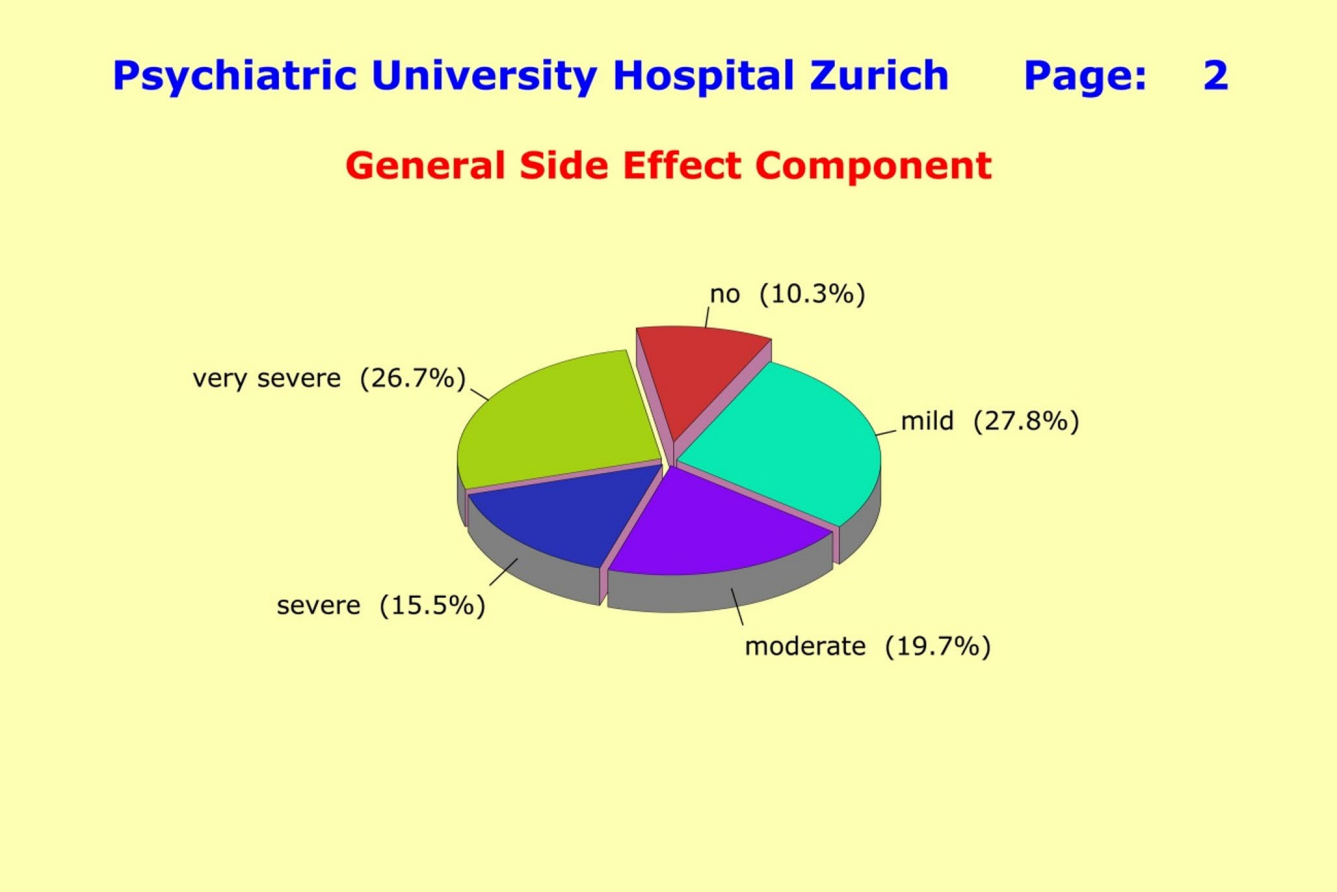
Splitting the side effects into an unspecific “*General Side Effect Component*” and “*Treatment-specific Side Effect Components*” revealed considerable differences in terms of clinical severity. For the general component, 61.9% of patients reported moderate to very severe side effects (26.7% very severe), versus 68.1% with moderate to very severe side effects (37.1% very severe) for the treatment-specific component. The high proportion of very severe side effects should be a matter of serious concerns for the doctors in charge, as this can critically compromise the patients’ compliance with treatment

**Fig. 3:**
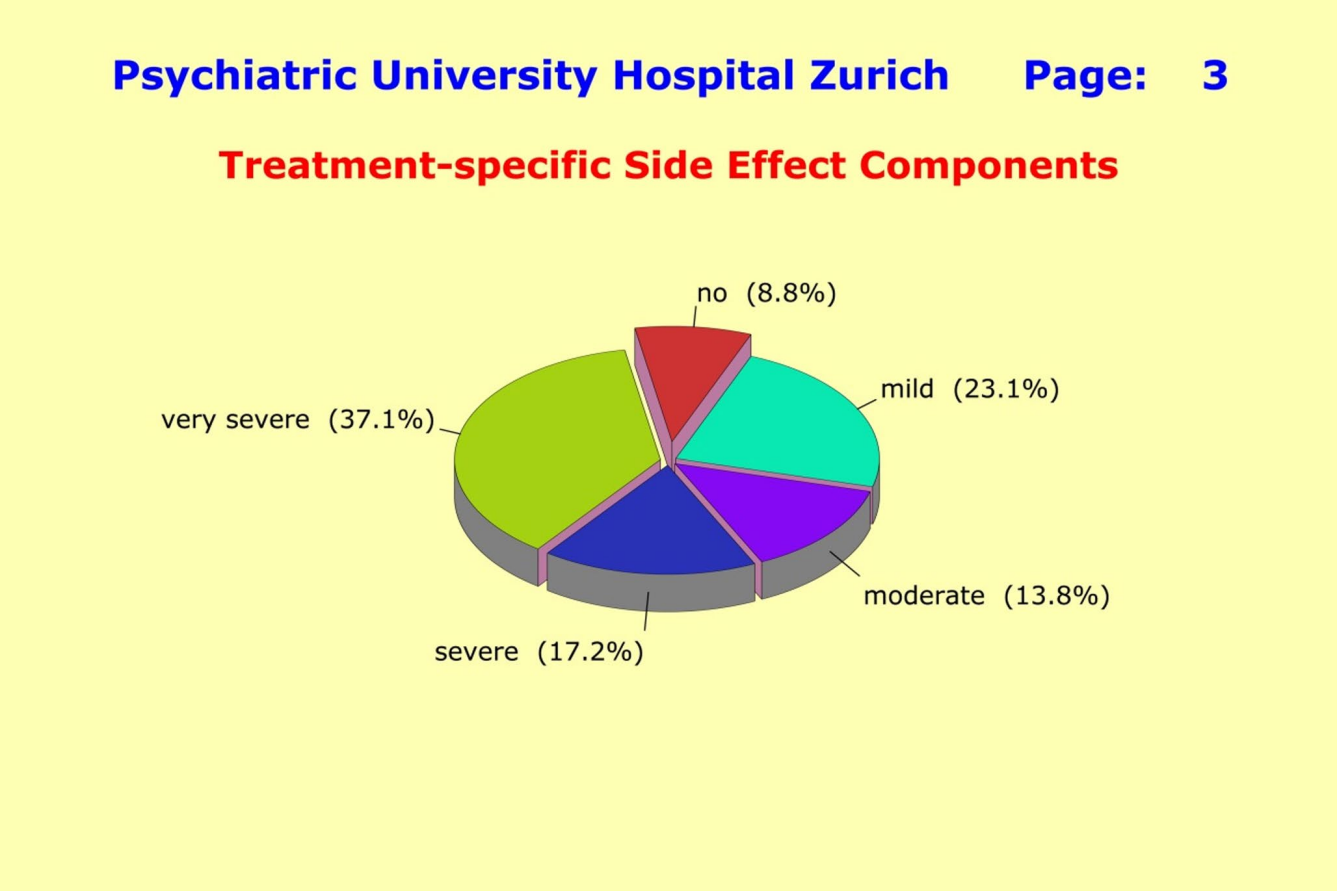
Splitting the side effects into an unspecific “*General Side Effect Component*” and “*Treatment-specific Side Effect Components*” revealed considerable differences in terms of clinical severity. For the treatment-specific component, 68.1% of patients reported moderate to very severe side effects (37.1% very severe), versus 61.9% with moderate to very severe side effects (26.7% very severe) for the general component. The high proportion of very severe side effects should be a matter of serious concerns for the doctors in charge, as this can critically compromise the patients’ compliance with treatment

The unspecific general component was quantified through a single sum score «G*SIDE*», whereas the treatment-specific components entered the subsequent analyses in their original form as «*SEX*», «*AUTO*», «*NEURO*», and «*CARDIO*». Splitting the side effects into an unspecific general component and drug-dependent components revealed considerable differences in terms of clinical severity: for the general component, 61.9% of patients reported moderate to very severe side effects (26.7% very severe) (Fig. 2); while for the treatment-specific components, 68.1% of patients report moderate to very severe side effects (37.1% very severe) (Fig. 3). The high proportion of very severe side effects must be a matter of major concerns for the doctors in charge, as this can critically compromise compliance with treatment.

Generalized linear regression models (“*GLMs”*) were carried out separately for the side effect components under investigation in order to estimate the amount of variance that was explainable through the variables age, gender, diagnostic group, symptom severity at baseline, body weight at baseline, onset of improvement, and number of concurrent drugs. The number of concurrent medications «*NDRUG*s» was found to be, by far, the most determining factor across all side effect components (r = 0.2544; p < 0.0001).

«*NDRUG*s» was largely independent of age (*p* = 0.0483), gender (*p* = 0.7731), and severity at baseline (*p* = 0.9376). However, there was a tendency that F2 patients with *milder* symptoms received *more* concurrent medications (*r*=-0.0932; *p* = 0.0129). Also, patients with a higher baseline weight received more concurrent medications (*r* = 0.1195; *p* = 0.0015).

The generalized linear regression models explained between 10% (*CARDIO*: R-Square = 0.0868) and 30% (*GSIDE*: R-Square = 0.2905) of the observed variance. Expectedly, by using non-linear models (such as NNs), it was readily possible to increase the explained variance by an average of additional 20% under the constraint of reproducibility, but without providing new insights.

As to the treatment-specific components «*SEX*», «*AUTO*», «*NEURO*», and «*CARDIO*», we attempted to identify those drugs (or combinations of drugs) that contributed most to the development and progression of the treatment-specific side effects. However, the sheer number of medications used by the treating psychiatrists of this study, —summing up to an average of 4.50 ± 2.68 concurrent medications per patient, and amalgamated in numerous combinations—, has led to an enormous diversity of medication regimens. Even though some drug combinations were more common in the multitude of medication regimens, a clear and widely accepted treatment strategy was not discernible. As a result, we were not successful in identifying single drugs that consistently explained a major proportion of the observed variation in side effect patterns under polypharmacy. In this context, it is worth noting that patients receiving a similar or even the identical medication “load” can display quite different side effect patterns, both in terms of the type of symptoms they experience as well as their clinical severity.

### Unwanted weight gain

**Fig. 4:**
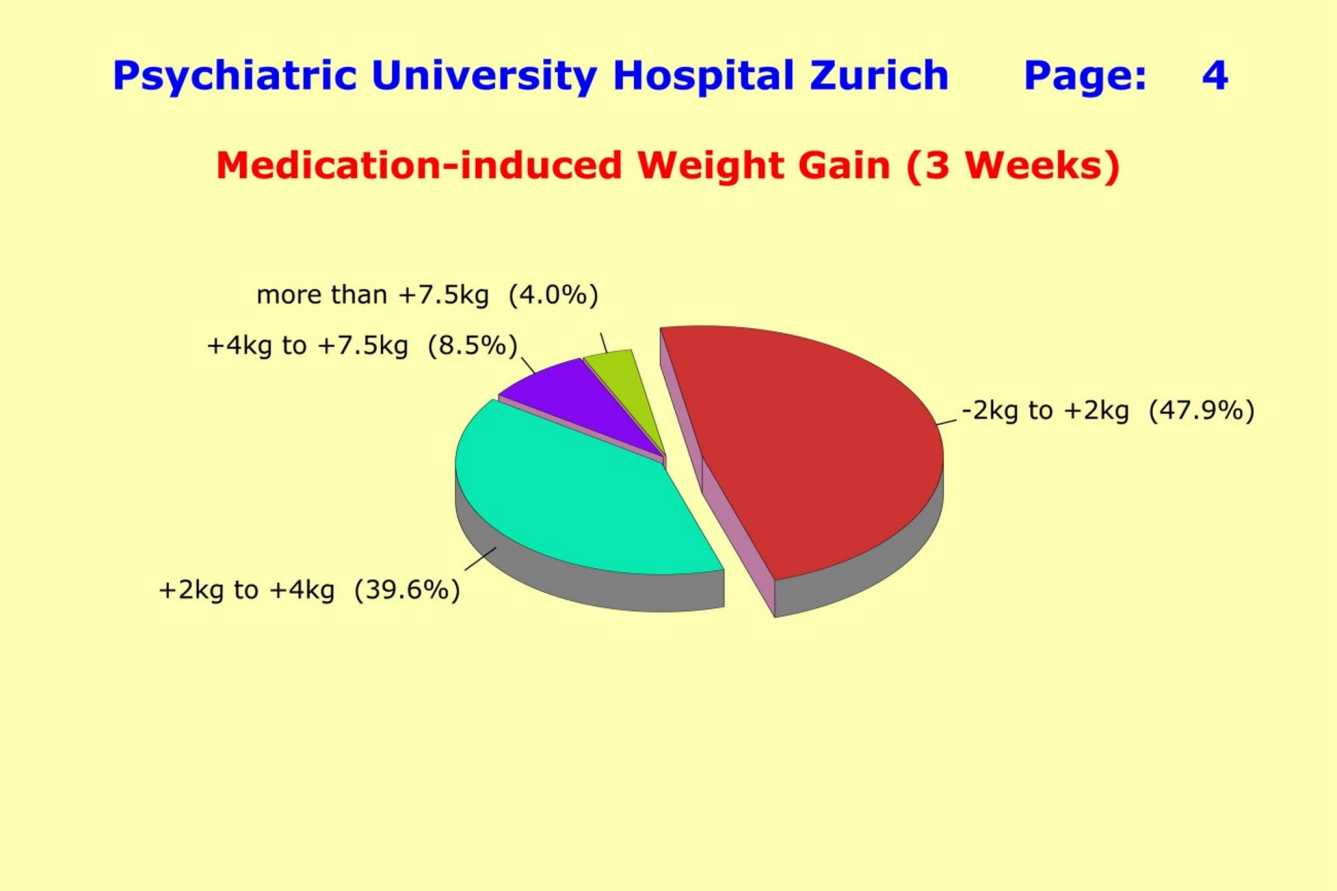
Of the 491 patients with full weight change trajectories, less than half remained within ± 2 kg of their initial weight (47.9%) throughout the observation period of 3 weeks (< 5% with a weight loss of up to 2 kg; a weight loss > 2 kg was not observed). The other half of the patients (52.1%) experienced significant weight gains: 39.6% in the range of 2 kg to 4 kg, 8.5% between 4 kg and 7.5 kg, and 4.0% of more than 7.5 kg. This weight gain was largely independent of the patients’ primary diagnosis

Of the 491 patients with full weight change trajectories, less than half remained within ± 2 kg of their initial weight (47.9%) throughout the observation period of 3 weeks (< 5% with a weight loss of up to 2 kg; a weight loss > 2 kg was not observed). The other half of the patients (52.1%) experienced significant weight gains: 39.6% in the range of 2 kg to 4 kg, 8.5% between 4 kg and 7.5 kg, and 4.0% of more than 7.5 kg (Fig. 4).

This weight gain was largely independent of the patients’ primary diagnosis (F2 vs. F3 patients: 2.36 ± 2.28 kg vs. 2.40 ± 2.27; *p* = 0.5577 [***t*** = 0.587; ***df*** = 489]). Even though male patients generally showed somewhat higher weight gains than female patients, the respective differences did not reach statistical significance (*p* = 0.1145; [***t***-test]), and there was no significant age dependence either.

Subsequently, a generalized linear regression model of unwanted weight gain was carried out in order to estimate the amount of variance that was explainable through the variables age, gender, diagnostic group, symptom severity at baseline, body weight at baseline, onset of improvement, and number of concurrent drugs. Although the variables number of concurrent drugs (*p* = 0.0002), body weight at baseline (*p* = 0.0370), age (*p* = 0.0327) and gender (*p* = 0.0597) reached statistical significance, the total variance explained by the model remained pretty low overall (R-Square = 0.1085; *p* = 0.0422). However, the majority of patients reported an increased appetite from the first day of treatment (*r* = 0.2752; *p* < 0.0001), making this symptom a valuable indicator for urgent clinical countermeasures against medication-induced weight gain.

## Results: genotype level

The results on the phenotype level supported the proposed model that the side effects under polypharmacy are comprised of two parts: (1) an unspecific general component that evolves in a similar way across gender, diagnoses and substances, with severity being mainly determined by the patients’ overall medication “load”; and (2) a treatment-specific component that depends quite strongly on the chosen combination of substances and their interactions. In particular, the results on the phenotype level clearly supported the notion that the between-patient differences in side effect patterns are driven by genetic factors, given that patients receiving a similar or even the identical medication “load” can display quite different side effect patterns in terms of symptoms and clinical severity.

The molecular-genetic analyses relied on the following quantitative phenotypes: (1) the side-effect clusters; (2) the unspecific general side effect component «*GSIDE*», quantified through a single sum score; (3) the treatment-specific side effect components «*SEX*», «*AUTO*», «*NEURO*», and «*CARDIO*» in their original form; along with (4) the body weight «*WEIGHT*» at entry into study; and (5) the medication-induced unwanted weight gain «*WGAIN*». The analyses were guided by the results of our previous investigations into (1) the vulnerability of major psychiatric disorders where irregularities in genetic diversity revealed the existence of illness-specific genes for which certain genotypic patterns showed up exclusively in patients, but not in healthy controls [21]; and (2) the recovery among patients with schizophrenic disorders or major depression, where irregularities in genetic diversity revealed the existence of “response genes” that constituted a patient’s «recovery disposition» regarding therapy response [42].

Thus, we carried out the following steps: (1) we determined the genetic diversity inherent in the 902 study patients by evaluating the patients’ gene vectors constructed from the 549 genotyped SNPs; (2) we looked for genotypic patterns that were characteristic of the side effect phenotypes under investigation; (3) we looked for genotypic patterns that were characteristic of the weight-related phenotype; (4) we tried to identify “side effect genes” and “weight genes” as key elements for molecular-genetic neural net analyses; and (5) we run multilayer NNs to construct classifiers that predicted the side effect and weight phenotypes sufficiently well from the patients’ genotypic patterns.

### Genetic diversity

The evaluation of the gene vectors based on 549 genotyped SNPs resulted in a total of 7,748 different genotypic patterns, which was only one third (33.8%) of the theoretical number expected for this sample size under the assumption of uncorrelated SNPs. This indicated the existence of significant between-SNP correlations. In addition to the between-SNP correlations we also found between-gene correlations. In fact, genotypic patterns observed with a pair of genes must not necessarily be independent of each other but may be “linked” to some extent, so that genotypic patterns can co-occur more often than expected by chance, even across chromosomes. Almost one third of genes showed such correlations, with mean correlations around 0.107 ± 0.102 across diagnostic subgroups.

The number of different genotypic patterns per gene (“diversity index” ***d***) was found to be ***d*** = 80.5 on average (range 7-341), while being only weakly correlated with the constituent number of SNPs. A generalized linear regression model GLM of constituent SNPs explained no more than about 20% of the observed variance, whereas the combined factors chromosome, gene size, and gene position explained some 6%. All this suggested that genetic diversity most likely reflects an *intrinsic gene property that has emerged over the course of evolution*. It is worth noting that the diversity indices provide a direct measure of the genes’ information content regarding the resolution of between-subject differences: a diversity index of ***d*** = 7 indicates almost no between-subject variation (most subjects have identical genotypic patterns), whereas a diversity index of ***d*** = 341 means a high between-subject variation since there were 341 different genotypic patterns observed in the sample under investigation.

### Genotypic patterns involved in side effects and weight gain

In the next step, we used standard Artificial Intelligence (AI) procedures to screen the genes under investigation for multidimensional genotypic patterns that were present in the vast majority of patients with this phenotype, while, at the same time, were rare (< 10%) among patients without this phenotype, and vice versa. Genes that met these criteria (“*side effect genes*” or “*weight gain genes*”) received the highest weight in subsequent molecular-genetic NNs. As the patients’ side effect patterns showed major quantitative and qualitative differences between monotherapy and polypharmacy, they had to be analyzed separately.

Unexpectedly, the AI procedures failed to detect multidimensional genotypic patterns that discriminated between patients with moderate to severe side effects from patients with no or mild side effects. The AI analyses were carried out for all side-effect clusters with a particular focus on the treatment-specific clusters «*Sexuality*», «*Autonomic*», «*Neurological*», and «*Cardio-vascular*». Breaking down the data by gender and diagnosis did not change the general picture. This unsatisfactory outcome stands in clear contrast to our previous studies on (1) psychiatric vulnerability, and (2) recovery from psychiatric disorders under polypharmacy, where “singular genes” were found to feature multidimensional genotypic patterns that separated (1) patients from healthy controls, and (2) improvers from non-improvers quite well [21, 42].

Possible explanations for the unsatisfactory outcome could be (1) the clinically defined side-effect clusters do not constitute phenotypic entities (“phenotypes”) that are in sufficiently close interrelation with genotypic patterns (“linked to genotypes”); (2) there may exist genotypic heterogeneity such that *multiple pathways on the genotype level* lead to the *same clinical picture on the phenotype level*. For example, sleep disturbances may differ in their pathogenesis across patients, as could be the case for other clusters as well; and (3) our set of psychiatry-centered candidate genes, which worked perfectly well in the context of (1) psychiatric vulnerability, and (2) recovery from psychiatric disorders, did not include the genes that are specifically involved in the pathogenesis of adverse side effects under polypharmacy.

Next, using *all* available multidimensional genotypic patterns (rather than patterns pre-selected by AI as contributing to the separation of phenotypes), we trained molecular-genetic NNs that aimed at separating patients with no or mild side effects from patients with moderate to severe side effects, while simultaneously discriminating between side-effect clusters. Again, focus was laid on the treatment-specific clusters «*Sexuality*», «*Autonomic*», «*Neurological*», and «*Cardio-vascular*». Here, too, the analyses did not yield clinically useful models for any of the phenotypes, as false-negative classification errors < 15% under the constraint of reproducibility were always combined with false-positive classification errors > 50%. Breaking down the data by gender did not lead to reasonable results either. This latter outcome, however, could be expected given the results presented in Table 1.

Next we addressed the question of the extent to which an unspecific genetic predisposition to developing adverse side effects under polypharmacy might explain the clinical data. In fact, polypharmacy with its conglomeration of various substances appears to induce a broad spectrum of bodily and psychophysiological reactions that affect all clusters in a way that the side effects of *all clusters increase simultaneously* when the number of concurrent drugs increases (the type of adverse reactions is presumably different for substances under monotherapy. However, side effects under monotherapy are typically less severe, and monotherapy has become obsolete in today’s psychiatry).

The phenotype to be linked to the hypothesized unspecific genetic predisposition was quantified through a single sum score «*GSIDE*». We then trained molecular-genetic NNs that aimed at separating patients with zero or mild *GSIDE* scores from patients with moderate to severe scores, using several different thresholds. For all chosen thresholds, models could be fitted to the empirical data so that the rate of false-negative classification errors fell well below 15%, —but at the price of a false-positive rate of over 50%. Likewise, for the 14 cytochromes examined (“CYPs”), including those with the most common oxidation pathways and associated with variable drug response (*CYP3A4*, *CYP3A5*, *CYP2C9*, *CYP2D6*, *CYP2C19* [48, 49]), no substantial interrelation with the *GSIDE* score could be found. Although some of the CYPs explained a few percent of the phenotypic variance, this was most likely due to sporadic signals with no clear biological significance, as is typically observed in such analyses.

As the analyses on the phenotype level clearly suggested the existence of treatment-specific side-effect clusters, the drug type could be expected to play a similarly crucial role regarding adverse side effects as in monotherapy. However, the ubiquitous polypharmacy approach in today’s psychiatry resulted in no less than 266 different drugs administered in 293 different combinations to the patients of this study. In consequence, we were not successful in identifying single drugs that consistently explained a major proportion of the observed variation in side effects under polypharmacy. And, there seems to be no viable way of linking certain side effects to a specific substance.

Only in the case of medication-induced weight gain, a few genes reached statistical significance without exhibiting, however, any major discriminatory power.

## Discussion

In a study of 902 inpatients treated for major depressive or schizophrenic disorders, we monitored in a very detailed way (1) medication regimens, (2) unwanted side effects, and (3) time course of improvement for up to 8 weeks. The primary goal was to develop a comprehensive quantitative side effect model that accounts for the various facets of clinically observable adverse events under polypharmacy treatment. Interest was focused on the clinical determinants of side effects as well as on the extent to which a genetically controlled “disposition” might enable, facilitate, impede, or prevent certain unwanted side effects.

A total of 9 side-effect clusters «*Sleep*», «*Appetite*», «*Sexuality*», «*Gastro-intestinal*», «*Cardiac-respiratory*», «*Autonomic*», «*Psychosomatic*», «*Neurological*» and «*Cardio-vascular*» were assessed through 48 items of the MEDIS instrument in order to detail the patients’ medication “load”. Our model followed the clinical observation that the side effects under polypharmacy treatments are comprised of two parts: (1) an unspecific medication-independent “general component” that evolves in a similar way across diagnoses and substances; and (2) a “medication-specific component” that depends quite strongly on the chosen combination of substances and their interactions.

The data of this study underlined the widespread clinical impression that unwanted side effects are a very common problem, closely linked to all forms of psychotropic medications. Between 61.9% and 68.1% of study patients reported moderate to very severe side effects (26.7–37.1% very severe), while response rates were with 29.5–35.7% quite modest. More than half of the patients (52.1%) experienced significant weight gains of 2 kg or more.

The data made it particularly clear that type and severity of side effects are mainly determined by the patients’ overall medication “load”. Consequently, patients under monotherapy experienced significantly fewer side effects than patients under polypharmacy. Most notably, response rates under polypharmacy were significantly lower compared to monotherapy. Increased side effect rates along with reduced response rates make it difficult to understand why polypharmacy has become today’s treatment regimen of choice for the vast majority of psychiatric patients.

The empirical data convincingly confirmed the proposed, clinically oriented side effect model. On the phenotype level, up to 30% of the observed variance could be “explained” by generalized linear regression models, with the dominating factor “number of concurrent drugs”. Attempts to link the medication-specific components «*SEX*», «*AUTO*», «*NEURO*», and «*CARDIO*» to single drugs were less successful, because of the sheer number of 266 drugs used by the treating psychiatrists in 293 different combinations, with an average of 4.50 ± 2.68 concurrent drugs per patient. Moreover, patients receiving a similar or even an identical medication “load” were found to display quite different side effect patterns, both in terms of the symptoms they experience and the overall severity.

The fact that patients can display quite different side effect patterns under identical medication “loads” pointed to the involvement of genetic factors in the development of adverse events under polypharmacy. To narrow down to the hypothesized genetic factors, this project did not rely on standard genetic association methods, nor did it aim to fit a causal polygenic model to the clinical data, when searching for genetically predisposed “side effect risks”. Rather, interest was focused on irregularities in genetic diversity which were recently found to be linked to a latent physical-genetic “recovery disposition” that controls recovery from major psychiatric disorders and initiates improvement by setting various thresholds for exogenous triggers. Accordingly, we analyzed the genetic diversity inherent in the study sample and looked for irregularities that might contribute to (1) a general unspecific predisposition to develop side effects under polypharmacy; (2) the development of treatment-specific side effects, such as neurological and cardio-vascular disturbances; and (3) the development of unwanted medication-induced weight gain. For this purpose, we relied on standard Artificial Intelligence (AI) procedures along with multilayer Neural Nets (NNs) and searched for combinations of multidimensional genotypic patterns that were characteristic of patients with side effects, while being rare (< 10%) among patients without side effects.

Contrary to expectations, these analyses failed to explain a clinically relevant proportion of the observed phenotypic variance. No side-effect-specific irregularities in genetic diversity could be detected, nor were attempts successful to classify side effect patterns by a combination of multidimensional “gene vectors” by means of NNs. Particularly disappointing was the fact that no notable link with the 14 cytochromes under investigation could be found, not even for the medication-induced neurological and cardio-vascular disturbances.

A possible explanation could be that the genetic predisposition to developing unwanted side effects is of a more quantitative nature, in the sense that a certain, individually different threshold has to be exceeded by the medication “load” in order to be noticeable by the patient. In other words, with the same genetic predisposition, the thresholds pertaining to polypharmacy may not be met under monotherapy. Hence, the subjective assessment of type and severity of side effects by the patients might be too unreliable to serve as phenotype for molecular-genetic analyses.

Another possible explanation for the failure of the analyses on the genotype level could be (1) there exists genotypic heterogeneity such that *multiple pathways on the genotype level* lead to the *same clinical picture on the phenotype level*. For example, sleep disturbances may differ in their pathogenesis across patients, as could be the case for other clusters as well; (2) our set of psychiatry-centered candidate genes, which worked perfectly well in the context of psychiatric vulnerability and recovery from psychiatric disorders, did not include the genes that are specifically involved in the pathogenesis of adverse side effects under polypharmacy; and (3) the genetic disposition to developing unwanted side effects under polypharmacy might be by far more complex than previously assumed, related to a completely different set of genes. This latter view gets support from the fact that the 14 cytochromes under investigation were found to play no more than a minor role, if at all.

In conclusion, a major step towards personalized medication in psychiatry is not in sight. Nonetheless, this study provided convincing evidence that side effects under polypharmacy are comprised of two parts: (1) a medication-independent genetic component that evolves in a similar way across gender, diagnoses and substances, with clinical severity being mainly determined by the patients’ overall medication “load”; and (2) a treatment-specific genetic component that depends quite strongly on the combination of substances and their interactions. While type and severity of side effects under polypharmacy are primarily determined by the overall medication “load”, the actually observed side effect patterns varied considerably between patients receiving the same medication “load”, thus stressing the role of genetics. However, our results suggested that the role of genetics in the development of side effects under polypharmacy is by far more complex than previously assumed, so that reliable predictions are difficult to accomplish. On the other hand, the patients’ side effects under polypharmacy can be reduced in a very straightforward way by simply reducing the medication “load” —which would presumably improve response rates as well.

## Conclusions

The empirical data confirmed the proposed, clinically oriented side effect model that the side effects under polypharmacy treatment regimens are comprised of two parts: (1) a medication-independent general component that evolves in a similar way across gender, diagnoses and substances; and (2) a treatment-specific component that depends quite strongly on the chosen combination of substances and their interactions. On the phenotype level, up to 30% of the observed variance could be “explained” by generalized linear regression models, with the dominating factor “number of concurrent drugs”. Attempts to link the medication-specific components to single drugs were less successful, because of the sheer number of medications used by the treating psychiatrists. On the genotype level, the analyses failed to explain a clinically relevant proportion of the phenotypic variance. In consequence, this study does not open a new way to a more personalized medication strategy. Given the more severe side effects combined with lower response rates observed under polypharmacy, however, clinicians should consider monotherapy and psychotherapy without supplemental psychotropic medication as valuable treatment options, in particular in the case of mildly to moderately ill patients.

### Limitations

The majority of patients came from Central Europe, so that the variation in biological ethnicity was modest. The molecular-genetic NN classifiers derived through this sample may not necessarily show the same performance with ethnically different populations.

**Table 1:** The percentages of patients who experienced moderate to severe adverse side effects revealed significant male-female differences (*p* = 0.0048; [***chi2*** = 22.1, ***df*** = 8])

Male-Female Differences Regarding Adverse Side Effects				
		Males	Females	
Neurological disturbances		12.1%	19.7%	
Autonomic disturbances		15.7%	25.3%	
Gastrointestinal disturbances		22.1%	36.5%	
Psychosomatic disturbances		22.1%	41.0%	
Cardiac-respiratory disturbances		32.9%	44.4%	
Appetite increased/decreased		47.9%	58.9%	
Sexuality		**52.1%**	**36.5%**	
Cardiovascular disturbances		61.4%	60.7%	
Sleep disturbances		65.7%	67.9%	

Adverse side effects were assessed by specifically trained health professionals on the basis of a catalog of 46 items covering 9 side-effect clusters (first column, sorted in ascending order). Adverse reactions were rated as *mild*, *moderate*, *severe*, or *very severe*. The given percentages relate to assessments during the first 3 days of treatment combined with assessments at the 10th day of treatment. There were only minor changes over time

**Table 2:**
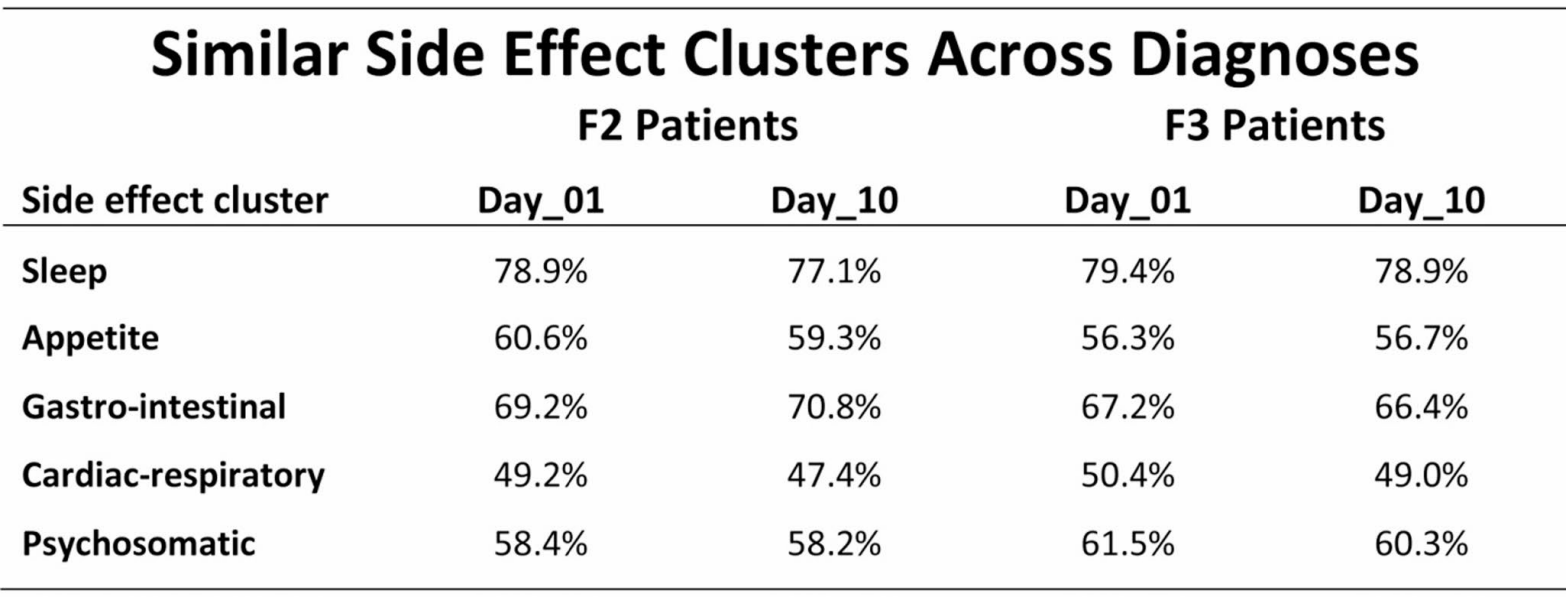
Similar side effect profiles across diagnoses and across treatment regimens for the five side effect clusters «*Sleep*», «*Appetite*», «*Gastro-intestinal*», «*Cardiac-respiratory*», and «*Psychosomatic*» suggested the existence of a general, non-medication-specific predisposition to developing unwanted side effects

The side effect clusters were assessed through 25 items of the MEDIS instrument and developed from the very beginning of medication. The clinical severity of all five clusters was only slightly reduced after 10 days of treatment

**Table 3:**
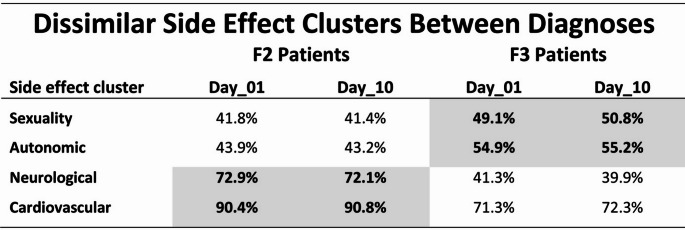
The four side effect clusters «*Sexuality*», «*Autonomic*», «*Neurological*», and «*Cardiovascular*» displayed significant differences between diagnoses, presumably due to different medication priorities in the treatment of patients with F2 diagnoses (antipsychotic-oriented with antidepressants as co-medication) versus F3 diagnoses (antidepressant-oriented with antipsychotics as co-medication)

The side effect clusters were assessed by 21 items of the MEDIS instrument and developed from the very beginning of medication. The clinical severity of the clusters was quite stable over time and seemed to be virtually unchanged on “*Day 10*” of the study when compared with the first assessment “*Day 01*”

## Electronic supplementary material

Below is the link to the electronic supplementary material.

Supplementary Material 1

Supplementary Material 2

## Footnotes

1 Sustained: no deterioration beyond 15% of achieved baseline score reduction.

2 The use of ***relative*** weight gain in [%] would have meant that overweight people with a 5 kg weight gain still fall into the group of patients without significant change, thus downplaying medication-induced weight gain in an inappropriate way.

## Declarations

### Availability of Data and Material

The data on which this study is based are not publicly available due to the sensitivity of molecular-genetic material, which does not allow adequate protection of personal information.
